# Are There Biological Correlates of Response to Yoga-Based Interventions in Depression? A Critical Scoping Review

**DOI:** 10.3390/brainsci14060543

**Published:** 2024-05-25

**Authors:** Ravi Philip Rajkumar

**Affiliations:** Department of Psychiatry, Jawaharlal Institute of Postgraduate Medical Education and Research (JIPMER), Pondicherry 605 006, India; jd0422@jipmer.ac.in; Tel.: +91-413-2296280

**Keywords:** major depressive disorder, yoga, complementary and alternative medicine, hypothalamic–pituitary–adrenal axis, biomarker, interleukin-6, brain-derived neurotrophic factor

## Abstract

Depression is the most common mental disorder worldwide. Both antidepressants and psychotherapy are effective in treating depression, but the response to these treatments is often incomplete. Yoga-based interventions (YBIs) have been advocated by some researchers as a promising form of alternative treatment for depression. Recent research has attempted to identify the biological mechanisms associated with the antidepressant actions of YBIs. In this scoping review, conducted according to the PRISMA-ScR guidelines, the PubMed and Scopus databases were searched to retrieve research on biomarkers of response to YBIs in patients with depression. These studies were also critically reviewed to evaluate their methodological quality and any sources of bias. Nineteen studies were included in the review. Based on these studies, there is preliminary evidence that YBIs may be associated with increased serum brain-derived neurotrophic factor (BDNF) and reduced serum cortisol and interleukin-6 (IL-6) in patients with depression. However, many of these changes were also observed in the control arms, and the overall quality of the research was low. At present, it cannot be concluded that there are reliable biomarkers of response to YBIs in depression, though there are some potential biological correlates. Further advances in this field will depend critically on improvements in study design, particularly the minimization of sources of bias and the selection of more specific and sensitive biomarkers based on existing evidence from other treatment modalities.

## 1. Introduction

Depressive disorders are one of the leading causes of morbidity and disability worldwide [1]. This group of disorders is characterized by persistent low mood with associated changes in cognition, behavior, and biological functions. It includes major depressive disorder (MDD), characterized by discrete episodes of a greater severity, and dysthymia, characterized by chronic, “low-grade” depressive symptoms [2]. It is estimated that at least 300 million people suffer from depressive disorders globally; in the year 2017 alone, it was estimated that there were around 250 million new cases or episodes of depression [3]. Depression arises from a complex interplay of biological, psychological, and social factors, which results in significant cross-national and cross-regional variations in its occurrence. It is estimated that the prevalence of depression is 4.1–5.8% in European countries, 5.9% in the United States, 3.4–4.8% in Africa, and 3–4.5% in Asian countries. In India, the prevalence of depression is estimated at 4.5% [3].

The treatment of depressive disorders usually involves pharmacological treatment with antidepressant medications and psychotherapies such as interpersonal therapy (IPT) and cognitive behavioral therapy (CBT) [4]. Though these treatments are effective, there is a significant number of patients who do not respond to them. It is estimated that at least 30–40% of patients will not respond to initial treatment with antidepressants, and the likelihood of response to subsequent treatments is of a similar order [5,6,7,8]. Moreover, some patients who respond initially to medication may relapse despite continued treatment [9]. Psychotherapies have advantages over medication in terms of effectiveness, safety, and sustained response [10], but may not be readily available in low-resource settings [11]. There is significant heterogeneity within the group of depressive disorders, and patients with different symptom profiles may respond better to specific types of treatment or combinations of treatments [12].

The limitations of conventional monotherapies for depression were highlighted by a recent meta-analysis, which showed that combined therapies were superior both in terms of efficacy and of sustained improvement in symptoms of depression [13]. However, providing combined pharmacotherapy and psychotherapy poses its own challenges in terms of requirements in time, personnel, and infrastructure.

The increasing awareness of the limits of “conventional” treatment for depression has led to a renewed interest in complementary and alternative therapies. This term “complementary and alternative medicine” (CAM) or “integrative medicine” encompasses a wide range of interventions that fall outside the umbrella of “allopathic” medicine [14]. A wide range of CAM-based treatments have been tested in depression, but only a few have shown replicated evidence of efficacy [15,16]. Most of these treatments have been used as adjuncts to standard treatments, though some have been tried as monotherapies for milder forms of depression [17].

### 1.1. Yoga as a Treatment for Depression

Yoga is an ancient spiritual discipline originating from India. The word *yoga* is derived from a Sanskrit root meaning “union”, which refers to the aim of this discipline—to achieve union between individual and universal (divine) consciousness, and between mind and body [18]. The disciplines that constitute yoga can be broadly divided into four components: physical movements (*asana*), relaxation (*savasana*), breathing techniques (*pranayama*), and meditative techniques (*dhyana*) [19]. Yoga is sometimes classed together with CAM or integrative therapies. Experts in the field emphasize that it is not primarily a “treatment” or an “intervention” for physical or mental illnesses; rather, it is a set of practices aimed at mind–body “oneness” or “integration” [20,21]. Nevertheless, some researchers have investigated the potential of yoga as a treatment for mental disorders, including depressive disorders. The first clinical trials in this field, conducted over two decades ago, found that yoga was effective in the treatment of both major depression and dysthymia, with an efficacy comparable to that of antidepressant monotherapy. However, these reports came from a single research center in India and required replication [22,23].

Subsequent studies have tended to confirm this result, with some caveats. A systematic review of yoga for depression, published in 2017, concluded that it did have positive effects that exceeded those of placebo. This review also identified significant methodological concerns in existing research, pertaining to study power, differences between monotherapy and combined therapy trials, and a lack of medium- or long-term results. These limitations reduced the confidence that could be placed in “positive” research reports [24]. A more recent meta-analysis, published in 2023, found that yoga had a moderate effect in reducing symptoms of depression (Cohen’s *d* = −0.60 to −0.64) and a small effect on associated symptoms of anxiety (Cohen’s *d* = −0.26). The authors of this review cautioned that many of the included studies had a significant risk of bias. They concluded that overall quality of evidence for yoga in depression was “low to moderate” in quality as per the Grading of Recommendations Assessment, Development, and Evaluation (GRADE) guidelines. Another limitation of this review is that it included some studies in which depression was only a secondary outcome, and in which patients did not fulfill diagnostic criteria for MDD [25].

Results from the most recent published reports suggest that yoga, when used as an adjunct or “add-on” to treatment as usual (TAU), may be effective and cost-effective in the management of depressive episodes. Such results have been obtained both from India [26,27] and from high-income European countries [28,29]. However, it is unclear if consideration of these subsequent results would substantially alter the findings of the most recent meta-analysis.

As a result, expert recommendations for the treatment of depression have taken varying positions on the value of yoga, with no clear consensus [30,31]. The conclusion reached by the Indian Psychiatric Society (IPS) is particularly relevant in this context, as India is the country in which most clinical trials of yoga for MDD have been conducted. The IPS guideline concludes, “Studies related to role of the traditional therapies like meditation, yoga and related techniques have been mostly published in documents of various organizations propagating that particular technique. Well-designed scientific studies to authenticate these claims need to be conducted” [32].

### 1.2. Can Biomarkers Be Used to Improve the Quality of Yoga Research in Depression?

#### 1.2.1. Limitations of Clinical Research Pertaining to Yoga in Depression

Based on the current evidence, it can be tentatively concluded that YBIs are effective in reducing depressive symptoms in some patients, but that these results cannot be readily generalized due to methodological limitations, including the possibility of bias. In a review addressing this issue, Nauphal et al. critically evaluated the limitations and outlined an agenda for future research [33]. They identified five questions that needed to be addressed by researchers before firm recommendations regarding yoga could be incorporated into standard practice. These questions can be briefly summarized las follows:

- Are specific types of YBIs better than others in the treatment of depression? This is important because different authors have used different yoga-based techniques or modules, with different admixtures of some or all of the domains of yoga [19]. Variations in the exact type of intervention can lead to significant variability in observed outcomes [30].
- How often/for how long should YBIs be used to achieve a meaningful response? Most available research has only assessed short-term response over a period of 8 weeks or less, but depression is a chronic and recurrent illness, and it is not clear if brief YBIs have a sustained effect [34].
- Are there any adverse effects associated with the use of YBIs in depression? One of the “selling points” of YBIs is that they are safer than pharmacological treatments. However, there have been reports of adverse events, such as paradoxical increases in anxiety or depression, particularly with the use of meditation-based techniques [35,36].
- How do YBIs synergize with “standard” treatments for depression? Several clinical trials in this field have involved the comparison of adjunctive YBIs to “treatment as usual” in patients receiving recommended doses of antidepressants. However, it is unknown if the observed effects of YBIs in this context are simply additive, or if they enhance the effects of prior treatment. It is also unknown if YBIs are superior to other forms of adjunctive treatment, such as pharmacological augmentation or combined pharmacotherapy and psychotherapy [37].
- What are the barriers and challenges in accessing YBIs for patients in diverse settings? Several factors could potentially reduce the acceptability of YBIs, though they have not been specifically studied in depression. These include physical pain or disability, varying cultural attitudes towards the acceptability of yoga, a lack of qualified trainers or therapists, and financial constraints [38].

To some extent, these questions are being answered by recent research. For example, there is now more evidence on the combination of YBIs with standard therapy than there was in 2019 [26,27,28,29,39,40]. We know that a wide range of YBIs show comparable efficacy in depression, including hatha yoga, Sudharshan Kriya Yoga (SKY), body-oriented yoga, mindful yoga, and even yoga packages developed specifically for depression [39,40,41]. However, there have been no head-to-head comparisons of specific YBI techniques or modules, or attempts to identify relationships between specific techniques and improvements in specific symptoms of depression.

It is possible that controlled clinical trials with a better design and a reduced risk of bias could provide more definitive evidence on the role of YBIs in depression. However, even if evidence of clinical improvement is demonstrated, the mechanisms through which such improvement occurs are unknown. There is an unanswered, sixth question that remains to be addressed: What are the molecular and cellular processes associated with the response to yoga in depression? Depression is a complex syndrome associated with alterations in central nervous, autonomic, neuroendocrine, immune-inflammatory, and gut–brain axis functioning [42,43]. Distinct yoga techniques can potentially exert beneficial effects on pathways implicated in the pathogenesis of depression. For example, relaxation and breathing techniques may ameliorate the neuroendocrine dysregulation seen in depression [44], while meditation may have beneficial effects on the functioning of specific regions of the cerebral cortex [45]. There is a need to understand and characterize these mechanisms, so that improvements in symptoms can be correlated with underlying physiological and biochemical changes [46]. Understanding these changes at the molecular, cellular, and organ level would have three key benefits [47]:

- A better understanding of the biological correlates of symptom improvement in depression.
- A more evidence-based approach to YBI, facilitating a rapprochement between yoga practitioners and practitioners of modern medicine, thereby avoiding the extremes of skepticism and unrealistic claims [32].
- A personalized approach to the use of YBI in depression, through the identification of those patients who would respond best to them.

#### 1.2.2. Biomarkers and Their Subtypes

In other words, what is required is a careful study of biological correlates, and if possible of *biomarkers*, associated with response to yoga-based interventions in depression. A biomarker has been defined by the United States Food and Drug Administration (FDA) and National Institutes of Health (NIH) Biomarker Working Group as “A defined characteristic that is measured as an indicator of normal biological processes, pathogenic processes or responses to an exposure or intervention” [48]. Biomarkers are measures based on “molecular, histologic, radiographic or physiologic characteristics”, and are distinct from measures of an individual’s emotions, behavior, or responses to a questionnaire [49]. They are objective measures of cellular, organ, or system function that can be correlated with changes in self- or clinician-rated (subjective) status. Characterization of a biomarker requires a description of its identity, biological plausibility, and measurement method [48].

Biomarkers have been subtyped into five broad categories: diagnostic, monitoring, response, predictive, and prognostic [48,49]. A brief description of these subtypes is provided in Table 1.

#### 1.2.3. Biomarkers Related to Depression and Its Treatment

There is as yet no biomarker that can reliably diagnose depression [43]. However, several potential biomarkers have been evaluated for their utility in assessing disease activity, treatment response, and illness course in depression [50,51]. Response biomarkers for antidepressant therapies fall broadly into five categories: imaging, electrophysiology, cognition, biochemical, and genetic [52,53]. More recently, novel biomarkers such as levels of specific microRNAs or measures of DNA methylation have also been studied in relation to treatment response in depression [54]. In terms of the strength of evidence for each of these groups of biomarkers, large effect sizes (combined Hedge’s *d* > 1) have been noted for imaging, electrophysiology, cognitive and protein/nucleotide markers, and small to moderate effective sizes (combined Hedge’s *d* = 0.5) for genetic markers. However, marked inter-study variability has been observed in studies of electrophysiology, cognitive, and genetic biomarkers [52]. More details of these categories of biomarkers in depression research are provided in Table 2.

Most studies of treatment response in depression have evaluated biomarkers in relation to antidepressant drug therapy. However, these markers may also be useful in evaluating the response to other forms of treatment, such as electroconvulsive therapy [55], repetitive transcranial magnetic stimulation [56], and psychotherapies such as cognitive behavioral therapy (CBT) [57].

#### 1.2.4. The Difference between “Biological Correlates” and “Biomarkers”

In this context, it is important to note that several studies have examined changes in the levels of other molecules implicated in the pathogenesis of depression, such as pro-inflammatory cytokines and other inflammatory mediators, in relation to antidepressant response. Though some experts consider these valuable predictors of treatment response [58], others differ with this opinion and prefer to label them “biological correlates” instead of “predictors” or “biomarkers” [52]. This distinction is important, because not all biological changes seen in the course of treatment for depression correlate directly with the response. Moreover, levels of some of these proteins or hormones may remain elevated even after successful treatment. Such biochemical parameters cannot be truly called “biomarkers” [48]. The controversy over whether changes in cytokines are “response” or “predictive” biomarkers in relation to treatment in depression is unresolved; as of now, the consensus is that these molecules are biological correlates that may prove to be biomarkers pending future research [50,59]. This distinction will prove to be important when evaluating claimed “biomarkers” of response to YBIs.

#### 1.2.5. Possible Biological Correlates of Yoga and Their Relevance to Depression

Experts in the field of yoga have claimed that its regular practice has beneficial effects on a number of biological processes. However, as with yoga research in general, research on the “biology” or “neurobiology” of yoga has yielded inconsistent results, and is subject to significant methodological limitations [60]. With these caveats, it may be noted that recent meta-analyses suggest that yoga may increase gray matter volumes in the insular and hippocampal cortices, improve functional connectivity in the prefrontal cortex and default mode networks, increase heart rate variability, and reduce levels of certain pro-inflammatory markers [61,62,63]. Theoretically, these changes could be beneficial for patients with depression [64]. This speculation could not be verified due to a lack of controlled clinical research on biological changes in patients with depression receiving YBIs. With the recent growth of research in this area, it is important to critically review the evidence that has now accumulated, and to see if it can bring a greater degree of objectivity to the use of YBIs in clinical practice.

The aims of this review are fourfold:

- to summarize the current state of research on possible biological correlates associated with yoga-based interventions in patients with depression;
- to assess whether any of these correlates qualify as biomarkers, using the rigorous definitions provided by the FDA-NIH guidelines and earlier researchers;
- to critically evaluate the quality of this research, with a particular focus on possible sources of bias;
- to situate these findings in the broader context of biomarker research related to treatment response in depression in general.

## 2. Review Process

The objective of the current paper is to review existing studies of potential biomarkers associated with response to YBI in patients with depression. Due to the heterogeneity of the available research, a scoping review methodology was adopted based on the PRISMA-ScR guidelines [65]. The completed PRISMA-ScR checklist for this review is available as Supplementary Material Table S1.

For this review, the PubMed and Scopus databases were searched for relevant papers from a scoping perspective. All relevant papers published up to March 2024 were included. The search strategy was constructed based on both the PRISMA-ScR guidelines and prior scoping reviews of biomarker research in psychiatry [65,66,67]. In the first step of this review, a broad search strategy was used to retrieve citations related to the broad area of yoga-based interventions for depression. The following terms were used: (“major depression” OR “depressive episode” OR “major depressive episode” OR “depressive disorder” OR “major depressive disorder” OR “recurrent depressive disorder” OR “dysthymia”) AND (“yoga” OR “hatha yoga” OR “yoga-based intervention” OR “yoga-based therapy” OR “yoga therapy”), included in either the title, abstract, or full text.

A total of 495 citations were retrieved at this step. After removal of duplicate citations and screening out of unrelated papers based on the title and abstract, 187 full-text articles were screened for inclusion of the scoping review. As one of these articles had been retracted due to errors in reporting the results, 186 full texts were reviewed. In the second step, full-text articles were screened for suitability for the review. Studies were selected if they fulfilled the following criteria:

- Clinical trials in patients with depression, defined as major depressive disorder or dysthymia, diagnosed using the standard diagnostic criteria of the American Psychiatric Association or World Health Organization.
- No primary medical comorbidity, such as diabetes mellitus, cancer, or cardiovascular disease.
- Use of a yoga-based intervention, either as a monotherapy or as an adjunct to standard treatment or “treatment as usual” (TAU).
- Assessment of response to treatment in terms of improvements in depressive symptoms using a standardized and validated rating scale, such as the Hamilton Depression Rating Scale (HDRS) or Montgomery–Asberg Depression Rating Scale (MADRS).
- Measurement of one or more potential biomarkers at baseline and/or during treatment. Biomarkers were selected only if they fulfilled the criteria of conceptual clarity and biological plausibility. To establish *conceptual clarity*, the definitions of “response” or “predictive” biomarkers were used as per the FDA-NIH criteria [48]. To establish *biological plausibility*, only those molecules that had been identified as markers or predictors of treatment response in depression in previous systematic reviews and meta-analyses were included [50,51,52,53,54].
- Papers published in English.

The following types of studies were excluded:

- Trials of patients with other medical conditions (e.g., fibromyalgia, pre-menstrual symptoms) in which depressive symptoms were measured as a secondary outcome.
- Trials in which information on reductions in depressive symptoms was not reported.
- Studies whose duration was too brief to meaningfully assess treatment response in depression. Most trials of any intervention of depression require a period of 4 to 8 weeks to observe a response; therefore, trials with a duration <4 weeks were not included in the review.

**Figure 1:**
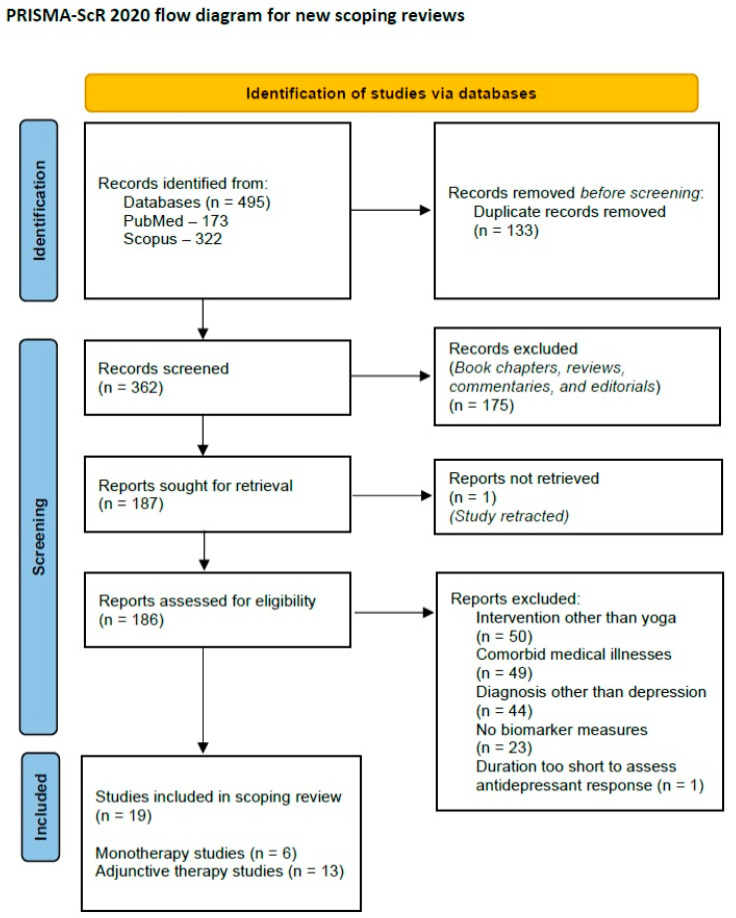
PRISMA-ScR 2020 flow diagram for the current review.

Based on these criteria, a total of nineteen papers were selected for inclusion in the review. A PRISMA-ScR flow diagram depicting the review process is provided below (Figure 1).

At the third step, data from each paper were extracted and tabulated under the following headings:

- Study sample characteristics: country of origin, setting, sample size;
- Clinical diagnosis, including any comorbidities if documented;
- Treatment(s) received in addition to YBI, if specified;
- YBI characteristics: type of YBI, number of sessions;
- Biomarker types and measures;
- Study results, both positive and negative, in terms of changes in biomarkers and/or associations between biomarkers and treatment response;
- Whether the given biological correlate could be considered a possible biomarker, based on the FDA-NIH criteria.

The fourth step involved assessment of study quality. In view of the concerns raised regarding the methodological quality of research on YBIs for depression, two aspects of study quality were assessed for each paper. First, each study was assessed using the BIOCROSS tool. This tool was specifically designed to evaluate the quality of biomarker studies in medical research [68]. It assigns a total score of 0 to 20 for a given paper, with higher scores indicative of better methodological quality. Second, the risk of bias in each of the biomarker-based clinical trials was assessed using the Cochrane Risk of Bias tool, version 2 (RoB 2) [69]. This is important because a high level of bias in the original trials could lead to false-positive findings regarding biomarkers of response to YBIs for depression. If the information in the retrieved reports was not sufficient for a complete RoB rating, the original published reports of the clinical trial were retrieved and data were extracted from them. This was necessary for two of the included studies [70,71].

## 3. Review Findings

### 3.1. Characteristics of the Included Studies

Overall, a total of six papers on YBI monotherapy [72,73,74,75,76,77] and thirteen papers on adjunctive YBI in patients with depression [78,79,80,81,82,83,84,85,86,87,88,89,90] were included in this review. After correcting for duplication of samples across some studies, data were available for 559 patients across 19 reports. The complete details of all included publications are provided in Table 3.

Among these papers, sixteen included patients with MDD, one included patients with either MDD or dysthymia, one included patients with dysthymia alone, and one included patients with either MDD or anxiety disorders with significant depressive symptoms. In all but one of the studies of MDD, patients were diagnosed based on standard diagnostic criteria, namely the American Psychiatric Association’s Diagnostic and Statistical Manual (DSM-IV/DSM-5) or the World Health Organization’s International Classification of Diseases and Disorders (ICD-10).

The majority of published reports (14 of 19) were from India [72,73,75,77,78,79,80,81,82,84,86,87,88,90]. Nine of these fourteen reports were from a single Indian center [72,73,77,81,82,84,87,88,90]. There were three publications from the United States [74,76], one from Germany [83], and one from Italy [85].

### 3.2. Study Quality

Study quality was assessed using the BIOCROSS and RoB 2 instruments. The mean BIOCROSS score for the papers included in this study was 13.4 ± 3.3, indicating a generally acceptable methodological quality. The lowest score obtained was 8 (out of a maximum of 20) [72,77] and the highest was 19 [83]. Studies obtained high mean scores on assessments of study hypothesis (1.6 of a maximum of 2) and description of the biomarkers measured (1.6). The lowest scores were obtained for limitations (1.0), statistical modeling of biomarker changes in relation to clinical response (1.1), and statistical analysis in general (1.2).

In other words, most studies described their rationale in clear and logical terms, and used valid and replicable laboratory methods to estimate potential biomarkers. However, they had deficiencies in terms of statistical analysis, modelling biomarker changes in relation to therapeutic response, and of emphasizing study strengths and positive findings without fully acknowledging limitations.

When assessed for risk of bias, sixteen of the nineteen included studies were found to be at high risk of bias [72,73,74,76,77,78,79,80,81,82,83,84,85,86,87,90], and three had some concerns pertaining to bias [75,88,89]. None of the studies fell into the low-risk category. On average, studies were found to be at high risk of bias in a median of two domains. When considering the individual domains of the RoB 2, only one study showed evidence of bias arising from a possible deviation in protocol [72]. However, five studies had concerns related to missing outcomes, eight studies had concerns related to the allocation of subjects to groups, eight had concerns related to the selection of the result, and nine had potential biases related to outcome measurement.

Overall, bias was a significant issue of concern in the existing literature. There was no significant difference in the proportion of studies rated at high risk of bias between monotherapy and adjunctive studies (5/6 vs. 11/13; *p* = 1.000, Fisher’s exact test) and between studies from India and those from other countries (12/14 vs. 4/5; *p* = 1.000, Fisher’s exact test).

In addition to these findings, study sample sizes were low (mean 39.1 ± 24.7 participants, range 15–84), and none of the reports included a formal sample size estimation. This suggests that this research may have been statistically underpowered in general.

### 3.3. Biological Correlates, Symptom Severity, and Treatment Characteristics

A wide range of potential biomarkers were studied in relation to adjunctive yoga in the existing literature. Based on the taxonomy suggested by Voegeli et al. for biomarker studies of treatment response in depression, these could be classified as follows: electrophysiology (*n* = 8), biochemical (*n* = 7), cognitive (*n* = 2), neuroimaging (*n* = 1), and genetic (*n* = 1). Electrophysiological and biochemical markers were the most frequently assessed, while neuroimaging and genetic markers were rarely evaluated [52]. In two studies, biomarkers were estimated only at baseline [73,74]; in the remainder, they were measured both at baseline and after the completion of treatment.

In most studies (17 out of 19), symptom severity was measured using a standardized rating scale for depressive symptoms. The most commonly used instrument was the Hamilton Rating Scale for Depression (HAM-D), followed by the Beck Depression Inventory (BDI). A single study used the Quick Inventory of Depressive Symptomatology (QIDS). Two studies did not report symptom severity at baseline [77,87]. Among the seventeen studies using a rating scale, two used a HAM-D cutoff of 18, indicating moderate depression, to select patients, but did not provide mean or median symptom scores. In the remaining studies, mean symptom scores for MDD ranged from 17.6 to 24.6 on the HAM-D and 23.2 to 30.8 on the BDI, indicating moderate to severe depression.

Among studies of electrophysiological markers, four studies examined changes in brain electrical activity using electroencephalography (EEG) [72,73,77] or transcranial magnetic stimulation (TMS) [83]; the remaining four evaluated cardiac autonomic functioning [74,80,85,88]. Biochemical studies showed marked heterogeneity, including assessments of neuroendocrine functioning [83,84,86], immune-inflammatory markers [86,89,90], brain-derived neurotrophic factor (BDNF) [82,86], and oxidative stress [86].

The type and duration of yoga-based interventions varied significantly across studies. Techniques used included Iyengar yoga [74,76], Sahaj yoga meditation [78,79,80], Hatha yoga [83,89], a depression-specific yoga module developed by experts [91], Sudarshan Kriya yoga [72,73,85], and a yoga- and meditation-based lifestyle intervention [75,86]. The duration of treatment ranged from 2 to 12 weeks, though most studies provided yoga-based interventions for 8–12 weeks. Details of these types of YBI, and which of the four components of yoga were included in each of them, are provided in Table 4. All interventions included at least two of the components of yoga, and three of them included all four components—movement, breathing, relaxation, and meditation. The most frequently used YBI was the yoga therapy module for depression, which was used in seven studies, all from the same center [91].

In studies of adjunctive YBIs, concurrent treatments are an important confounding factor. Twelve of the thirteen studies of adjunctive therapy included patients with antidepressants “as usual”, meaning that they were allowed to continue stable doses of antidepressant treatment in a naturalistic manner. One study attempted to standardize pharmacological treatment by randomizing participants to receive either the antidepressant escitalopram (10 mg/day) or the atypical antipsychotic quetiapine (300 mg/day) [83]. In twelve of the studies, adjunctive yoga was compared to pharmacotherapy alone, which served as the control group. In a single report from the United States, the control group received an educational intervention on lifestyle modification in addition to medication [89].

### 3.4. Biological Correlates vs. Biomarkers of Yoga-Based Intervention

The body of research included in this review yielded mixed results in terms of changes in possible biomarkers related to yoga-based interventions. Twelve studies reported findings that could be interpreted as positive, six reported no significant effects of YBI on the specified biomarker, and one reported a trend towards a possible effect of yoga. The interpretation of these findings is complicated by the fact that very few studies directly modelled the statistical relationship between changes in biomarkers and changes in depressive symptoms. Four studies tested for such a relationship [82,83,85,86] but only two established a possible link with the YBI of interest [82,86]. Two studies reported a temporal association between biomarker changes and symptom improvement, but did not explore this further [72,77]. This reflects the deficiencies in statistical analysis identified by the BIOCROSS ratings, as mentioned in Section 3.2.

When applying the criteria for biomarkers of response based on the FDA-NIH definition, more significant concerns were identified. Of the nineteen papers reviewed, twelve (63%) did not satisfy the definition for a response or predictive biomarker. In other words, any findings reported in these papers can only be considered “biological correlates” of YBIs in depression and not biomarkers. Four papers [75,83,86,90] reported results that could be considered response biomarkers, and two [74,81] reported findings that were suggestive but not conclusive. Overall, only four of fourteen Indian studies (28.6%) and two of five studies from other countries (40%) attempted to check for genuine “biomarker” effects. This difference was numerically in favor of the studies outside India, but was not statistically significant (*p* > 0.5, Fisher’s exact test). The existing research could be best described as identifying biological correlates and not biomarkers of response to YBIs in patients with depression. Detailed study results are given in full in Table 3, with key findings discussed below.

#### 3.4.1. Electrophysiological Studies

Among electrophysiological studies that involved measures of brain activity, a single study found that an adjunctive YBI module increased the cortical silent period. An association with symptom change was not reported [87]. Studies of electroencephalographic (EEG) parameters such as the P300 auditory event-related potential and alpha activity yielded inconsistent results [73,74,79].

Among studies of cardiac autonomic activity, a baseline component of heart rate variability (HRV) was associated with a better response to Iyengar yoga monotherapy [74], but studies of changes in HRV-related autonomic parameters following treatment yielded equivocal results [80,85,88].

All electrophysiological studies were assessed as being at a high risk of bias affecting study outcomes, except for a single study of heart rate variability [88].

#### 3.4.2. Biochemical Studies

*a. Neurotrophic factors:* Three studies, one of monotherapy and two of adjunctive therapy, examined changes in BDNF in relation to YBIs for depression [77,81,86]. All three reported increases in serum BDNF after YBI. In one study, this change was also observed in patients receiving TAU [81]. Two of these studies reported a correlation between increased BDNF and improvement in depressive symptoms [81,86].

*b. Immune-inflammatory mediators:* Two studies reported a reduction in peripheral levels of pro-inflammatory cytokine IL-6 after adjunctive YBI [86,89], and one reported a reduction in the complement components C1q, Factor H, and properdin [90]. These changes were not significantly correlated with improvements in depression.

*c. Neuroendocrine correlates:* Two studies of adjunctive YBIs found a reduction in serum cortisol after treatment with yoga, but not TAU. The relationship of these changes to the response was unclear [84,86]. In contrast, a study of Hatha yoga found no effect of yoga on cortisol responses to dexamethasone or CRH stimulation. In the latter study, normalization of the cortisol response was associated with improvement in depression in both the yoga and TAU groups [83].

*d. Others:* A single study found that adjunctive YBI, but not TAU, was associated with reductions in markers of oxidative stress and cellular aging. These changes were not significantly associated with treatment response [86].

All these studies, except one study of pro-inflammatory cytokines [89], were rated as being at high risk of bias.

#### 3.4.3. Neurocognitive, Genetic, and Imaging Studies

*a. Neurocognition:* A study of Sahaj yoga meditation found significant improvement in two tests of sustained attention in patients receiving yoga compared to TAU [78]. A study of an adjunctive YBI module found improvements in tests of concentration, processing speed, and verbal memory in both the yoga and TAU groups [82]. The relationship between these changes and treatment response was not studied. Both these studies were rated as having a high risk of bias.

*b. Genetics:* A single study examined the effect of serotonin transporter (*SLC6A4)* and methylene tetrahydrofolate reductase (*MTHFR)* functional polymorphisms on response to yoga-based monotherapy. No association was found between either variant and response to yoga, though *SLC6A4* genotype was associated with antidepressant response [75]. This study was rated as having some concerns with regards to bias.

*c. Neuroimaging:* A single study found evidence of increased thalamic GABA following Iyengar yoga monotherapy over 12 weeks on magnetic resonance spectroscopy; this was not clearly related to treatment response [76]. This study was rated as having a high risk of bias.

## 4. Critical Analysis of the Review Findings

Yoga-based interventions (YBIs) have been advocated for by some researchers and experts as an emerging option for the treatment of depression. There has been a significant growth of clinical research in this field. Results of YBI monotherapy have been mixed, though there is substantial evidence that adjunctive YBI outperforms patients receiving treatment as usual, and is well-tolerated. The mechanisms underlying this possible therapeutic effect are largely unknown, and have been investigated only recently. It is possible that some of the biological pathways influenced by YBIs are similar to those implicated in the action of antidepressants [64]. However, as will be seen below, such a conclusion cannot yet be drawn confidently due to certain conceptual and methodological concerns.

### 4.1. Replicated Findings Pertaining to Biological Correlates of YBI Response in Depression

A wide range of putative biomarkers have been investigated in relation to YBIs for patients with depression. As seen in Section 3.3, the majority of these do not satisfy strict criteria for biomarkers of treatment response. Moreover, replication of findings is an important concern in biomarker research in depression, and findings from single studies must be interpreted with prudence [52]. If we confine ourselves to results that have been replicated, or demonstrated by at least two independent groups of researchers, then only three findings emerge as being of potential relevance: increased serum BDNF [77,81,86], reduced serum cortisol [84,86], and reduced serum IL-6 [86,89].

These findings are potentially relevant, because alterations in all these three biochemical markers have been implicated in the pathophysiology of depression and in the response to conventional antidepressant treatments. Depressive episodes are associated with reduced peripheral BDNF [92], increased peripheral IL-6 [93,94], and dysregulated functioning of the HPA axis [95,96]. To some extent, these alterations have been found to normalize after successful treatment with medications, psychotherapy, or electroconvulsive therapy [97,98,99], though it should be noted that none of these have been consistently identified as biomarkers of treatment response in depression [52].

In addition, the biological processes indexed by these molecules are intimately linked to each other in the genesis of depressive disorders. Early childhood adversity, chronic stress, or depression itself can lead to HPA axis dysregulation, associated with increased cortisol levels and dexamethasone non-suppression [100,101]. These changes in HPA axis responsiveness lead to an increase in peripheral inflammation, as evidenced by increased peripheral levels of cytokines such as IL-6 [102]. Increases in peripheral inflammation can trigger microglial activation and neuroinflammation, leading to reduced neural plasticity, which is correlated with reduced levels of BDNF and with depressive symptomatology [103,104].

Additional support for a possible beneficial effect of YBIs on these molecular pathways comes from studies of yoga in healthy controls and in patients with disorders other than depression. In these patients, the practice of yoga was also associated with apparently beneficial reductions in peripheral pro-inflammatory cytokine and cortisol levels [105,106,107]. However, as noted in a recent meta-analysis, changes in these markers are not always statistically significant, and their clinical relevance is open to question [63].

The convergence of these three lines of evidence suggests that the effects of YBIs on HPA axis functioning, reduced peripheral inflammation, and increased neurotrophic factor production may be related to their benefits in patients with depression. However, even this conclusion is only provisional, as most of these studies did not directly model the relationship between changes in the potential biomarkers and reductions in depressive symptoms. In one study, there appeared to be a significant association between increased BDNF and treatment response [86], but this was not consistent across studies [81].

Findings related to other biological correlates, such as measures of oxidative stress, cerebral cortical inhibition, heart rate variability and brain GABA levels, cannot be definitively interpreted due to the limited quantity and quality of evidence available. There is evidence that these processes are dysregulated in patients with depression, lending some support to these findings [108,109,110,111]. Among these results, perhaps the most plausible are those related to HRV. This parameter, which is a measure of the balance between cardiac parasympathetic and sympathetic activity, is reduced in depression and may predict response to antidepressants [110]. Moreover, the practice of yoga may increase HRV in healthy individuals [112]. Though it is possible that HRV may prove to be a useful marker response to YBIs in depression, the inconsistency and low quality of the available evidence precludes such a conclusion at the moment [74,85,88].

### 4.2. Correlation or Causation?

The key question to be considered when appraising the results of biomarker studies is whether changes in specific biological processes are meaningfully associated with changes in the patient’s symptomatic status. Stated in simple terms, it is necessary not just to show that yoga changes levels of certain well-defined markers, but that these changes correlate with reductions in depressive symptoms. If this cannot be established, then one cannot speak of “biomarkers” in the most accurate sense, as explained in Section 1.2.5 and Section 3.3. In fact, accurate modeling of the relationships between biomarker levels and clinical status is one of the criteria for assessing the quality of biomarker research [48,64]. If biological parameters are altered in patients with depression, but do not correlate meaningfully with symptoms or their improvement, they can only be considered “disease markers” but not response biomarkers as per the FDA-NIH guidelines [48,52]. As noted in Section 3.3, only four of the studies attempted to model a possible causal relationship between changes in biomarkers and symptomatic improvement, and this was tentatively confirmed in two of these studies, both involving BDNF [82,86]. Two further studies reported a temporal association between biomarker changes and clinical response to YBIs. While temporal association is necessary to establish that a given marker is a response biomarker, it is not sufficient, as certain parameters may change contemporaneously with clinical improvement without being meaningfully linked to it. For example, treatment with antipsychotics can lead to objective, measurable increases in body weight and metabolic parameters in patients that are concurrent with clinical response. However, these increases occur regardless of patient diagnosis, and are not meaningfully connected with contemporary models of the pathogenesis of psychosis. Therefore, they are not biologically plausible biomarkers of antipsychotic response [113].

In conclusion, the existing research cannot confirm a causal relationship between any particular biomarker—with the possible exception of serum BDNF—and response to YBIs in depression. This limitation, which arises from shortcomings in study design and data analysis (Section 3.2), needs to be addressed in future research in this field.

### 4.3. Limitations of the Existing Research

Apart from concerns related to replication and causality, there are certain important limitations of the existing research. These should be considered when interpreting the results of individual studies, and are enumerated below.

- Biomarker selection: Recent reviews of biomarker research in depression have highlighted the limitations of certain biological correlates—particularly electrophysiological and biochemical measures—as genuine markers of treatment response. Experts in the field believe that there is a need to move beyond these biomarkers and to investigate novel predictors of treatment response in depression, such as measures of DNA methylation or protein expression, in combination with digital methods of phenotyping and machine learning [114,115,116,117]. In contrast, most studies of YBI in depression have used biological correlates that have already been extensively investigated and found to be of limited specificity and predictive power [52,56,92]. Similarly, though neuroimaging and cognitive findings have yielded the best effect sizes for prediction of antidepressant response [52], only three of the studies reviewed here made use of these markers [76,80,82]. In addition, some studies have used markers that are not of proven value in predicting treatment response in depression, such as heart rate or EEG alpha activity [79,80]. Results from such research, even if positive, are of limited value.
- Secondary analyses and multiple comparisons: Some of the studies presented in this paper are based on re-analyses or secondary analyses of original trial data [78,79,80,81,82,84,87,88]. Such secondary analyses introduce a significant risk of false positive results, as they are not based on analysis of a pre-planned primary biomarker outcome [118]. Likewise, some of the included studies have analyzed a large number of previously untested biomarkers. Though this approach may be useful in generating novel hypotheses [119], it increases the possibility of false positive findings, especially if appropriate statistical corrections are not made [120]. For example, one of the included studies examined multiple markers of neuroplasticity, oxidative stress, cellular aging, and neural plasticity [86]; if twenty such markers are evaluated, at least one false positive can be expected at *p* < 0.05 [121]. As a result, even the positive findings identified in this review should be viewed with caution.
- Participant-related biases: The majority of included studies are from India, where yoga has its historical and spiritual origin. The practice of yoga in Indian patients is imbued with cultural meanings that extend beyond its use as an “antidepressant” treatment [122]. Such factors may affect patients’ preferences for YBIs, leading to biases in sample selection even when patients’ depression severity scores appear comparable. Moreover, when these patients practice a YBI for depression, they may experience an accentuated “meaning response”, similar to that observed when receiving placebo treatments in a drug trial. This can produce biochemical and physiological changes that are not specifically related to yoga [123].
- Confounding effects of variations in yoga technique: As illustrated in Table 4, the YBIs used by different groups of researchers vary widely. Though they may have certain key components or principles in common, the intensity at which these components are administered, and their relative emphasis in each treatment “module” or “package”, vary widely. Moreover, there are variations in therapist skill and experience and patient adherence to home-based practice that are difficult to quantify. As a result of this, a positive finding obtained in one study cannot be generalized to clinical practice if the YBI differs substantially. Thus, for example, some studies of the “yoga therapy module for depression” found evidence of an increase in BDNF following treatment [77,82]; however, it cannot be concluded that the same effect will be observed in patients receiving Hatha yoga or Sudarshan Kriya Yoga. This is an important limitation of yoga research in depression in general [33,124].
- Effects of other confounders on biological correlates: As noted in (a) above, most of the putative biomarkers studied in relation to YBIs for depression are non-specific in nature. This means, inter alia, that their levels can be altered by a number of factors not directly related either to depression or to yoga. Such confounding factors include age, gender, levels of physical activity, dietary practices, obesity, exposure to psychosocial adversity during childhood, ongoing stressors, and comorbid medical illnesses [94,125,126]. The studies reviewed in this paper have made little or no attempt to model for the possible effect of these confounders; as a result, it is difficult to conclude that either the levels of these biomolecules, or changes in them, are specifically linked to the presumed benefits of YBIs.
- Low methodological quality: As can be seen from the BIOCROSS and RoB 2 assessments of individual studies, particularly the latter (Section 3.2), the majority of included studies were at a high risk of bias, particularly with regards to outcome measures and their analysis. Moreover, statistical modeling of biomarker outcomes was often simplistic and could not establish a clear causal relationship between treatment and changes in biochemical or physiological parameters. Such evidence would receive an overall quality of “low” according to the GRADE guidelines [127], and cannot be reliably used to guide clinical practice.
- Lack of specificity of the observed changes to yoga: Even if the findings summarized in Table 3 can be established as biomarkers through further research, there is a key issue that remains unresolved—namely, the lack of specificity of such biological changes to yoga or YBIs per se. Similar changes in immune, autonomic, and endocrine parameters have been documented for other “body-oriented” or “mind-body” interventions, including exercise [128], meditation [129], relaxation techniques such as deep breathing or progressive muscle relaxation [130], and traditional practices from other countries [131]. Therefore, it is possible that the changes observed in YBI research are non-specific concomitants of participation in several meditation- or movement-based therapies. As there are no head-to-head trials comparing changes in biological parameters in patients receiving YBI and those receiving these treatments, this question remains unresolved.
- Country of origin effects: It has been known for at least the past two decades that the country of origin is an important confounder when evaluating the “positivity” of study outcomes. For example, studies of acupuncture from China or Japan are more likely to yield positive outcomes than those from other countries. This may reflect both participant biases, as mentioned in (c) above, and implicit biases on the part of researchers and regional journals [132]. In the case of yoga, it has been documented that studies from India are up to 25 times more likely to be positive than those from other countries [133]. A subsequent systematic review involving a larger number of studies found that this was probably due to selection bias [134]. While the measurement of biological parameters might appear to reduce this risk, it cannot negate it entirely, and it is possible that subtle biases may enter both during the study and in subsequent decisions to publish specific results [68,132,134]. Such methodological concerns have even been flagged by Indian researchers working in the field of complementary and alternative medicine [135]. This is a particular cause for concern in the current review, which is largely based on studies for India.

In conclusion, it can be stated that the overall quality of the evidence reviewed in this is low, and is subject to bias from multiple sources. Such evidence cannot be reliably used to predict treatment response in clinical practice. At best, it can be tentatively stated that YBIs for depression are accompanied by changes in certain biological processes, but that the significance of these changes remains unclear. There is an urgent need for better-designed studies with more rigorous statistical analyses and attempts to address potential sources of bias.

### 4.4. Synthesis and Future Directions

**Figure 2:**
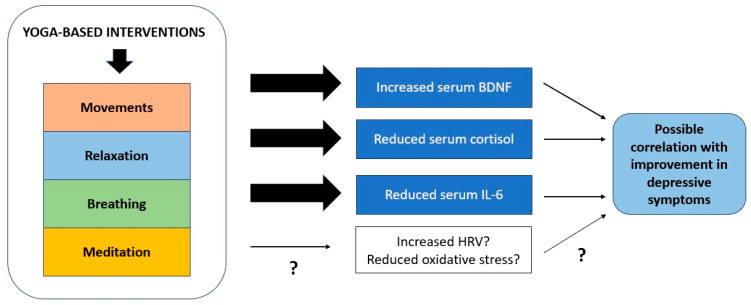
Mechanisms through which yoga-based interventions may influence biological processes involved in recovery from depression. “?” denotes that there is inconsistent or inadequate evidence to confirm the relevance of a particular process.

The existing research has uncovered a limited number of potential biological correlates of response to YBI. Though there are significant flaws in the underlying research; these changes can all be tentatively linked to a biological pathway that connects stress responses, peripheral inflammation, neuroinflammation, and neural plasticity. These mechanisms are illustrated graphically in Figure 2 below. This pathway has been implicated in the pathogenesis of depressive states by an extensive body of research, both in translational models and in patients with the disorder. It is possible that YBIs may have beneficial effects on depression through other pathways, including modulation of parasympathetic function and cortical inhibition, but these findings require replication. Moreover, none of these changes qualify as “biomarkers” using contemporary definitions, for the reasons summarized in Section 4.3.

The overall level of confidence that one can place in this evidence is low, due to various methodological limitations and sources of bias [136,137]. Therefore, the order of the day in this field would be to attempt to replicate these results in trials with a better design. This would include:

- Selection of study participants in such a way as to minimize bias, including multicentric trials with more sophisticated randomization methods.
- Comparative studies involving a YBI arm and a control arm receiving a different form of movement-related or mind–body therapy, such as exercise or meditation.
- Standardization of YBI modules and packages, to ensure comparability across studies, and to identify how specific yoga techniques affect discrete biological pathways.
- Selection of biochemical and physiological markers based on the most recent and consistent evidence from other studies of treatment response in depression. This would imply more research on neuroimaging and neurocognitive markers, as well as studies of novel biomarkers derived from epigenetic and proteomic research.
- Better handling of drop-outs, to avoid over-estimation of possible changes in biomarkers in patients receiving YBIs.
- Improved statistical analyses to minimize confounding effects and to reduce the likelihood of false positive findings.

Such improvements could be achieved through the development of standardized guidelines for conducting and reporting research on YBIs in depression, with reference to both biological correlates and clinical outcomes [138,139].

### 4.5. Strengths and Limitations of the Current Review

The chief strengths of this review are two-fold. First, it represents the first attempt to synthesize the existing evidence on the biological correlates of response to YBIs for depression. Second, it presents a critical analysis of several factors that could affect the quality of this evidence, and highlights areas where improvements or advances are crucially needed in future. For these reasons, it is of use both to clinicians with an interest in this form of therapy, and to researchers who wish to improve on existing research.

The limitations of this review should also be acknowledged. First, due to the heterogeneity of methods and outcomes, a formal systematic review or meta-analysis could not be carried out. Second, as only papers written in English were included, it is possible that relevant trials of YBIs written in other languages may have been omitted. Third, the study relied on citations retrieved from databases of biomedical research, in which studies of yoga may have been under-represented. Finally, it is possible that YBIs may improve depression through as yet unidentified biological mechanisms that were not considered in this review.

## 5. Conclusions

Based on the existing evidence, it is possible that biological mechanisms related to the stress response, immune-inflammatory activity, and neural plasticity may be involved in the beneficial effects of yoga-based interventions for patients with depression. Though these findings are plausible on the surface, they should be interpreted with caution due to several methodological shortcomings, and the overall quality of the available evidence is low. This review is a preliminary attempt at delineating both the biological processes that may underpin response to YBIs in depression, and the study-related errors and limitations that need to be addressed by future researchers in this field. The findings of this review are of potential interest both to clinicians involved in the evidence-based use of YBIs, and to researchers seeking a better understanding of biological changes associated with specific yoga techniques in patients with depression. Researchers in the field of yoga, particularly in those centers where research is most active, should view these results as a clarion call to improve the quality and replicability of their work. A key first step in this process is to identify and address the limitations of the existing research, and it is hoped that the data summarized and analyzed in this review will be of use to them in this endeavor.

## Figures and Tables

**Table 1:** Types of biomarkers in medical research, based on FDA-NIH guidelines.

Type	Definition [48]	Example
Diagnostic	A biomarker used to detect or confirm either a disease or a subtype of a disease.	Fasting plasma glucose or glycosylated hemoglobin for diabetes mellitus.
Monitoring	A biomarker used to monitor the current status of either a medical condition or exposure to a drug or environmental agent.	Hepatitis C ribonucleic acid (HCV-RNA) level to assess disease activity in hepatitis C.
Response	A biomarker used to show that a given biological process has occurred in a person exposed to a drug or environmental agent	International normalized ratio (INR) to assess the pharmacological action of warfarin
Predictive	A biomarker used to identify which individuals will respond favorably or unfavorably to a given drug or environmental agent	Estrogen receptor positivity to predict response to estrogen receptor antagonism in breast cancer
Prognostic	A biomarker used to predict the likelihood of disease recurrence or progression in patients already diagnosed with the disease	Prostate-specific antigen (PSA) to monitor possible cancer recurrence or progression after treatment of prostate cancer

**Table 2:** Biomarkers used to predict treatment response to antidepressants in patients with depression.

Category	Examples	Strength and Consistency of Evidence [52]
Neuroimaging	Hippocampal volume and prefrontal cortex grey matter volume on structural MRI Reduced amygdala activation at rest or during an emotional perception task on functional MRI	Moderate to large effect size; moderate variability
Electrophysiology	Changes in regional frequency spectra on EEG Measures of REM sleep latency and density on polysomnography	Small to large effect size; high variability
Cognition	Standardized tests of global cognitive functioning, processing speed, working memory, executive functioning	Large effect size; low variability
Biochemical	Measures of peripheral levels of hormones such as cortisol, neurotrophic factors such as BDNF, or immune-inflammatory mediators such as IL-6 Expression of specific genes such as *SLC6A4*, *BDNF*, and *TNFA* in peripheral blood	Moderate to large effect size; moderate variability
Genetic	Functional polymorphisms of the *SLC6A4*, *COMT*, *GRIK4* and *FKBP5* genes	Small to moderate effect size; high variability

**Abbreviations:** BDNF, brain-derived neurotrophic factor; *BDNF*, BDNF gene; *COMT*, catechol O-methyltransferase gene; EEG, electroencephalography; *FKBP5,* FK506 binding protein 5 gene; *GRIK4*, glutamate receptor, ionotropic, kainite 4 gene; MRI, magnetic resonance imaging; REM, rapid eye movement sleep; *SLC6A4*, serotonin transporter gene.

**Table 3:** Characteristics of studies examining biomarkers of response to yoga-based interventions in patients with depression.

Studies of Yoga-Based Interventions as Monotherapy										
Study	Country of Origin	Sample Size and Diagnosis	Yoga Intervention	Concurrent Treatment(s)	Category of Biological Correlate Studied	Biomarkers Estimated	Results	BIO-CROSS Score	Risk of Bias Assessment	Satisfies Definition of a Biomarker?
Murthy et al., 1997 [72]	India	*n* = 15, dysthymia (ICD-10 criteria); mean HAM-D 12.3 ± 3.2	Sudarshan Kriya Yoga (daily 30-min sessions over 12 months)	N/A	Electrophysiology	Auditory P300 event-related potential	Significant decrease in P300 amplitude but not latency after treatment; temporal association with clinical response	8	High risk	No
Murthy et al., 1998 [73]	India	*n* = 30; dysthymia, *n =* 15, MDD, *n* = 15 (ICD-10 criteria); mean HAM-D 12.3 ± 3.1 for dysthymia, 24.6 ± 6.3 for MDD	Sudarshan Kriya Yoga (daily 30-min sessions over 12 months)	N/A	Electrophysiology	Baseline auditory P300 event-related potential	No association between baseline P300 and response to yoga	10	High risk	No
Jain et al., 2014 [74]	United States	*n* = 16, MDD (DSM-IV criteria); mean HAM-D 12.9 ± 3.3	Iyengar yoga (12–13 sessions of 60 min over 8 weeks)	N/A	Electrophysiology	Baseline HRV parameters recorded through ECG	Lower HRV rVLF predicted better response to yoga	9	High risk	Possible
Tolahunase et al., 2018 [75]	India	*n* = 89, MDD (DSM-5 criteria); mean BDI-30.8 ± 8.6	Yoga-based lifestyle intervention (5 sessions/week over 12 weeks)	N/A	Genetic	Functional polymorphisms of *5-HTTLPR* and *MTHFR*	No association between either polymorphism and response to yoga	17	Some concerns	Yes
Streeter et al., 2020 [76]	United States	*n* = 28, MDD (DSM-IV criteria); mean BDI 26.1 ± 7.7	Iyengar yoga (12 weeks, randomized to low- or high-dose)	N/A	Neuroimaging	Thalamic GABA measured using MRS	Increased thalamic GABA low-dose yoga group; negative correlation between thalamic GABA and depression severity in high-dose group	15	High risk	No
Aditi Devi et al., 2023 [77]	India	*n* = 13, MDD (criteria not specified); mean symptom scores not given.	Depression-specific yoga module (10 sessions of 60 min over 2 weeks, home-based practice for 10 weeks)	N/A	Biochemical	Total, pro- and mature BDNF	Increased total and mature BDNF; reduced pro-/mature BDNF ratio; temporal association with clinical improvement	8	High risk	No
**Studies of Adjunctive Yoga-Based Interventions**										
**Study**	**Country of Origin**	**Sample Size and Diagnosis**	**Yoga Intervention**	**Concurrent Treatment(s)**	**Category of Biological Correlate Studied**	**Biomarkers Estimated**	**Results**	**BIO-CROSS Score**	**Risk of Bias Assessment**	**Satisfies Definition of a Biomarker?**
Sharma et al., 2006 [78]	India	*n* = 15, MDD (DSM-IV criteria); mean HAM-D 21.3 ± 4.3	Sahaj yoga meditation (8 weeks)	Antidepressants as usual	Neurocognitive	DST, LCT, TMT, RFFT	Greater improvement in LCT and DST-backward scores in yoga group. Correlation with clinical improvement not reported.	11	High risk	No
Sharma et al., 2007 [79]	India	*n* = 15, MDD (DSM-IV criteria); mean HAM-D 21.3 ± 4.3	Sahaj yoga meditation (8 weeks)	Antidepressants as usual	Electrophysiology	EEG alpha activity	No significant effect of yoga on alpha activity. Correlation with clinical improvement not reported.	11	High risk	No
Sharma et al., 2008 [80]	India	*n* = 15, MDD (DSM-IV criteria); mean HAM-D 21.3 ± 4.3	Sahaj yoga meditation (8 weeks)	Antidepressants as usual	Electrophysiology	Autonomic parameters—PR, RR, GSR	No significant effect of yoga on autonomic parameters. Correlation with clinical improvement not reported.	12	High risk	No
Naveen et al., 2013 [81,82] *	India	*n* = 35, MDD (DSM-IV criteria); mean HAM-D 17.6 ± 4.7	Depression-specific yoga module (12 weeks)	Antidepressants as usual	Biochemical (neurotrophic factor) Neurocognitive	Biochemical—Serum BDNF Neurocognitive—DST, TMT, RAVLT	Significant increase in BDNF and improvement in DST, TMT and RAVLT scores both in yoga and control groups; increased BDNF correlated with reduced depressive symptoms and improved TMA-A duration only in yoga group	16	High risk	Possible
Sarubin et al., 2014 [83]	Germany	*n* = 60, MDD (DSM-IV criteria); mean HAM-D 22.7 ± 6.5	Hatha yoga (1 h/week for 5 weeks)	Escitalopram (10 mg/day) or quetiapine (300 mg/day)	Biochemical (Neuroendocrine)	Cortisol response to serial DEX/CRH tests	No significant effect of yoga on cortisol responses. Changes in cortisol correlated with reduced depressive symptoms regardless of treatment.	19	High risk	Yes
Naveen et al., 2016 [84]	India	*n* = 35, MDD (DSM-IV criteria); mean HAM-D 17.6 ± 4.7	Depression-specific yoga module (12 weeks)	Antidepressants as usual	Biochemical (Neuroendocrine)	Serum cortisol	Significant reduction in serum cortisol in yoga group. Correlation with clinical improvement not reported.	15	High risk	No
Toschi-Dias et al., 2017 [85]	Italy	*n* = 46, “depression or anxiety disorders” (DSM-IV criteria); median HAM-D 20	Sudarshan Kriya Yoga (10 sessions over 2 weeks)	Antidepressants as usual	Electrophysiology	Cardiac autonomic parameters recorded through ECG	Reduced sympathetic modulation and improved parasympathetic modulation and cardiorespiratory coupling in yoga group. Temporal but not direct correlation with improvement in depressive symptoms.	13	High risk	No
Tolahunase et al., 2018 [86]	India	*n* = 58, MDD (DSM-IV criteria); mean BDI 23.2 ± 4.3	Yoga and meditation lifestyle intervention (12 weeks)	Antidepressants as usual	Biochemical (multiple)	Serum 8OH2dG, BDNF, cortisol, DHEAS, IL-6, oxidative stress markers (ROS, TAC), sirtuin-1, telomerase	Significant increase in BDNF, DHEAS, sirtuin-1 and telomerase and decrease in cortisol, IL-6, ROS, TAC and 8OH2dG in yoga group. Changes in BDNF correlated with reduction in depressive symptoms.	14	High risk	Yes
Bhargav et al., 2021 [87]	India	*n* = 70, MDD (DSM-IV criteria); mean symptom scores not given	Depression-specific yoga module (12 weeks)	Antidepressants as usual	Electrophysiology	TMS measures of cortical inhibition (CI, CSP, RMT, LICI, SICI)	Significant increase in CSP in yoga group. Correlation with clinical improvement not reported.	13	High risk	No
Gulati et al., 2021 [88]	India	*n* = 68, MDD (DSM-IV criteria); HAM-D cutoff ≥ 18	Depression-specific yoga module (12 weeks)	Antidepressants as usual	Electrophysiology	HRV parameters recorded through ECG	Trend towards greater decrease in HRF low-frequency/high-frequency ratio in yoga group; no significant group differences. Correlation with clinical improvement not reported.	18	Some concerns	No
Nugent et al., 2021 [89]	United States	*n* = 84, MDD (DSM-IV criteria); mean QIDS 12.7 ± 2.8	Hatha yoga vs Healthy Living Workshop (10 weeks)	Antidepressants as usual	Biochemical (immune-inflammatory)	Serum CRP, IL-6, TNF-α	Significant reduction in IL-6 in yoga group. Correlation with clinical improvement not reported.	17	Some concerns	No
Subbanna et al., 2021 [90]	India	*n* = 22, MDD (DSM-IV criteria); HAM-D cutoff ≥ 18	Depression-specific yoga module (12 weeks)	Antidepressants as usual	Biochemical (immune-inflammatory)	Plasma complement components C1q, C3, C3b/iC3b, C4, Factor B, Factor H, properdin	Significant reduction in C1q, Factor H and properdin in yoga group; not correlated with improvement in depression. Significant reduction in C4 in control group	13	High risk	Yes

* The findings of this study have been published across two reports. As they refer to the same sample of patients and outcome measurements, they have been presented together. **Abbreviations**: 8OH2dG, 8-hyrdoxy 2-deoxyguanosine; BDNF, brain-derived neurotrophic factor; CRH, corticotropin-releasing hormone; CRP, C-reactive protein; CSP, cortical silent period; DHEAS, dehydroepiandrosterone sulfate; DSM, Diagnostic and Statistical Manual for Mental Disorders; DST, Digit Span Test; DEX, dexamethasone; ECG, electrocardiography; EEG, electroencephalography; GSR, galvanic skin response; HRV, heart rate variability; IL-6, interleukin-6; LCT, letter cancellation test; LICI, long interval cortical inhibition; MDD, major depressive disorder; PR, pulse rate; RAVLT, Rey Auditory Verbal Learning Test; RFFT, Ruff Figural Fluency Test; RMT, resting motor threshold; ROS, reactive oxygen species; RR, respiratory rate; SICI, short interval cortical inhibition; TAC, total antioxidant capacity; TMS, transcranial magnetic stimulation; TMT, Trail Making Test; TNF-α, tumor necrosis factor alpha.

**Table 4:** Description of the different types of YBI used in the included studies.

Name	Brief Description	Movement	Relaxation	Breathing	Meditation
Hatha Yoga [83,89]	Sun salutation, postures, breathing exercises, relaxation, seated meditation	+	+	+	+
Iyengar Yoga [74,76]	Floor, sitting and standing poses, breathing exercises, intermittent relaxation	+	+	+	-
Sudarshana Kriya Yoga [72,73,85]	Daily practice of three specific breathing components	-	+	+	-
Sahaj Yoga [78,79,80]	Silent meditation and “thoughtless awareness” in a quiet room or with feet immersed in warm water	-	+	-	+
Yoga therapy module for depression [77,81,82,84,87,88,90]	Loosening exercises and postures, relaxation, breathing techniques, chanting meditation	+	+	+	+
Yoga-based lifestyle intervention [75,86]	Sun salutation, loosening exercises and postures, breathing exercises, relaxation, meditation with and without chanting	+	+	+	+

## Data Availability

No original data were generated for the purpose of this review.

## Supplementary Materials

The following supporting information can be downloaded at: https://www.mdpi.com/article/10.3390/brainsci14060543/s1, Table S1: PRISMA-ScR checklist for the current review.
